# Long-Term Outcomes and Imaging Characteristics of Patients with Scimitar Syndrome

**DOI:** 10.3390/jcdd13050196

**Published:** 2026-04-30

**Authors:** Hicran Gül Emral, Thao V. N. Nguyen, Ilaria Bo, Thomas Semple, Julene S. Carvalho, Piers E. F. Daubeney, Michael L. Rigby, Sylvia Krupickova

**Affiliations:** 1 Paediatric Cardiology Department, Royal Brompton Hospital, Guy’s and St Thomas’ NHS Foundation Trust, London SW3 6NP, UK; hicrangulseymen@hotmail.com (H.G.E.); vuthao3103@gmail.com (T.V.N.N.); ilaria.bo@nhs.net (I.B.); jcarvalho@nhs.net (J.S.C.); piers.daubeney@nhs.net (P.E.F.D.); michaellawrence.rigby@nhs.net (M.L.R.); 2 National Heart and Lung Institute, Imperial College London, London SW7 2AZ, UK; thomas.semple@nhs.net; 3 Radiology Department, Royal Brompton Hospital, Guy’s and St Thomas’ NHS Foundation Trust, London SW3 6NP, UK; 4 Cardiovascular and Genomics Research Institute, City St. George’s, University of London, London EC1V 0HB, UK

**Keywords:** scimitar syndrome, morphology, multimodality imaging, interventions, outcome

## Abstract

Scimitar syndrome is a rare congenital cardiopulmonary anomaly with marked anatomical and clinical heterogeneity, and long-term outcome data remain limited. We reviewed our single-center experience over a 33-year period. Patients evaluated between 1992 and 2025 were retrospectively analyzed. A total of 104 patients were included, with female predominance (63, 60.6%). The median age at first presentation was 0.4 years (IQR 0.0–16.7; range 1 day–68 years) with 59 patients (56.7%) presenting during infancy. At last follow-up, the median age was 18.5 years (IQR 8.7–30.6; range 60 days–70 years), with a median follow-up duration of 9.5 years (IQR 3.7–16.1). Dextrocardia was observed in 76 patients (73.1%). The most common associated defect was atrial septal defect (37 patients, 35.6%), while 23 patients (22.1%) had no additional cardiac defects. Respiratory manifestations predominated at presentation and follow-up. However, 18 patients (17.3%) were asymptomatic at diagnosis and 44 (42.3%) at follow-up. Partial anomalous pulmonary venous drainage was present in 87 patients (83.6%), and aorto-pulmonary collaterals in 70 (67.3%). Cardiac catheterization was performed in 78 patients (75.0%), and 47 (45.2%) underwent surgery. At last documented follow-up, 101 of 104 patients (97.1%) were alive, with three deaths occurring during follow-up.

## 1. Introduction

Scimitar syndrome is defined by anomalous pulmonary venous drainage, most commonly from the right lung, into a systemic venous structure, typically the inferior vena cava. It is frequently associated with a spectrum of cardiopulmonary abnormalities, including right lung hypoplasia, pulmonary artery hypoplasia, abnormal systemic arterial supply, and cardiac displacement. The condition represents a heterogeneous spectrum of anomalies rather than a single uniform entity [1,2]. The syndrome includes a broad spectrum of pulmonary and cardiovascular malformations which partially overlaps with pulmonary sequestration; therefore, the term venolobar syndrome has been used to encompass these associated anomalies [2,3].

Although scimitar syndrome has traditionally been categorized into infantile and childhood/adult forms based on age at presentation, clinical severity and outcomes are increasingly recognized to be driven primarily by the burden of associated cardiopulmonary anomalies and hemodynamic factors rather than age alone [1,4,5,6]. Furthermore, cases are being diagnosed antenatally.

The clinical spectrum ranges from patients presenting with respiratory distress, pulmonary hypertension, or heart failure to asymptomatic children and adults diagnosed incidentally during evaluation for murmurs or abnormal chest radiographs [7].

Scimitar syndrome encompasses right lung hypoplasia, hypoplasia or agenesis of the right pulmonary artery, abnormal systemic arterial supply most often arising from the descending aorta, and dextroposition of the heart secondary to reduced right lung volume. Additional congenital cardiac anomalies, particularly atrial septal defects, may coexist and further contribute to pulmonary overcirculation and pulmonary hypertension [8,9].

Management strategies for scimitar syndrome remain heterogeneous and controversial. While patients with mild shunting and preserved pulmonary hemodynamics may be managed conservatively, intervention is generally considered in the presence of heart failure, recurrent respiratory infections, pulmonary hypertension, or a significant left-to-right shunt (Qp/Qs >1.5–2.0) with evidence of right heart volume overload [1,9]. Cardiac catheterization plays a key role in the assessment of pulmonary hemodynamics and allows for transcatheter embolization of aortopulmonary collateral arteries, which may reduce pulmonary overcirculation and pulmonary artery pressure [9,10]. Surgical redirection of the scimitar vein to the left atrium has been performed using various techniques; however, postoperative pulmonary vein stenosis remains a major determinant of long-term morbidity and mortality [9,11].

Given the marked anatomical and clinical heterogeneity of scimitar syndrome and the lack of consensus regarding optimal timing and type of intervention, further data are required to better define prognostic factors and long-term outcomes. The aim of this study was to describe the anatomical spectrum, management strategies, and long-term outcomes of patients with scimitar syndrome in a large, single-center cohort over a 33-year period.

## 2. Methods

### 2.1. Study Design and Patient Population

This was a retrospective, single-center cohort study conducted at a tertiary referral center. Medical records of all patients diagnosed with scimitar syndrome and evaluated at our institution between 1992 and 2025 were reviewed. Patients referred from other institutions following initial assessment or treatment were also included, providing sufficient clinical and imaging data were available for comprehensive review.

### 2.2. Inclusion and Exclusion Criteria

Inclusion criteria for scimitar syndrome or a scimitar variant were defined as partial anomalous pulmonary venous drainage of the right or left lung to a systemic venous structure (inferior vena cava or IVC, IVC–right atrial junction, hepatic veins, azygos vein, superior vena cava (SVC), or to the right atrium).

The presence of an abdominal aorto-pulmonary collateral (APC), including pulmonary sequestration, was considered an inclusion feature only when occurring in association with scimitar syndrome or a recognized scimitar variant as part of the venolobar anomaly spectrum, and not as an isolated finding. 

Additional supporting anatomical features included right- or left-sided pulmonary artery or lung hypoplasia, abnormal pulmonary arterial branching patterns, and the presence of dextroposition.

Given the absence of universally accepted criteria for scimitar variants, patients were not subclassified into classical and variant forms for analytical purposes. Instead, a spectrum-based definition was adopted in accordance with previously published descriptions [12]. Patients with isolated aortopulmonary collaterals without associated anomalous pulmonary venous drainage were excluded. The diagnosis of scimitar syndrome was established based on multimodality imaging demonstrating anomalous pulmonary venous drainage to a systemic venous structure. This could be identified using non-invasive imaging modalities, including echocardiography, computed tomography (CT), or cardiovascular magnetic resonance (CMR), and/or invasive assessment with cardiac catheterization when performed. Invasive confirmation was not mandatory for diagnosis.

### 2.3. Data Collection

Clinical data were collected retrospectively from electronic and paper medical records. Extracted data included demographic characteristics, clinical presentation, outpatient and inpatient documentation, operative reports, and follow-up records. Imaging data from echocardiography, cardiac catheterization, cardiovascular magnetic resonance (CMR), computed tomography (CT), cardiopulmonary exercise testing (CPET), and spirometry were reviewed when available. Data were collected retrospectively from available records. Given the heterogeneity of the cohort and the longitudinal nature of follow-up, measurements (including Qp/Qs, CPET, and imaging data) were not restricted to a single standardized time point. When multiple assessments were available, clinically relevant data from initial evaluation, pre-intervention assessment, or follow-up were included, depending on availability.

### 2.4. Imaging and Hemodynamic Assessment

Imaging data were derived from clinical reports and, where necessary, were reviewed by two experienced pediatric cardiologists and one radiologist. The diagnostic findings reported in the Section 3 were based on multimodality imaging, including echocardiography, computed tomography (CT), cardiovascular magnetic resonance (CMR), and, where appropriate, cardiac catheterization. All imaging data were interpreted within the clinical context as part of routine practice, reflecting real-world management in a tertiary referral center.

#### 2.4.1. Echocardiography

Transthoracic echocardiography was used for initial diagnostic assessment and follow-up evaluation. Pulmonary venous drainage patterns, intracardiac anatomy, ventricular size and function, and Doppler assessment of pulmonary venous flow were evaluated according to standard practice and internal scanning protocols.

#### 2.4.2. Cardiac Catheterization

Cardiac catheterization was performed for diagnostic and/or interventional purposes. Hemodynamic measurements were obtained when clinically indicated, including intracardiac and great vessel pressures and calculation of Qp/Qs. Angiography was used to delineate pulmonary venous anatomy, systemic arterial collaterals, pulmonary arterial anatomy, and suspected venous obstruction.

#### 2.4.3. Cardiovascular Magnetic Resonance (CMR)

CMR was used for detailed anatomical and functional assessment, including ventricular volumes, ventricular function, shunt quantification, and pulmonary blood flow distribution. Ventricular volumes were indexed to body surface area, and Qp/Qs ratios were calculated using phase-contrast flow measurements.

#### 2.4.4. Computed Tomography (CT)

CT imaging was utilized for high-resolution anatomical assessment of pulmonary venous drainage, pulmonary venous stenosis, pulmonary artery anatomy, lung morphology, and associated thoracic abnormalities.

### 2.5. Definitions

#### 2.5.1. Pulmonary Venous and Scimitar Vein Obstruction

Pulmonary venous or scimitar vein obstruction was defined by one or more of the following criteria: non-phasic pulmonary venous flow or increased Doppler velocity on echocardiography, discrete anatomical narrowing identified on catheterization angiography, or venous stenosis demonstrated on cross-sectional imaging (CT or CMR).

Severity of obstruction was classified as mild or severe based on imaging characteristics and associated hemodynamic relevance.

#### 2.5.2. Pulmonary Artery Hypoplasia

Pulmonary artery hypoplasia was assessed based on relative vessel caliber compared with the contralateral pulmonary artery, using available imaging data (echocardiography, CT, or CMR) and clinical reports. Vessel diameters were recorded, and classification (mild vs. moderate-to-severe) was based on relative comparison and descriptive assessment as documented in clinical reports, reflecting real-world clinical practice in a retrospective cohort.

#### 2.5.3. Aorto-Pulmonary Collaterals

Aorto-pulmonary collaterals (APCs) were identified using cardiac catheterization angiography, CT, or CMR. Collateral size was classified as large or small based on relative vessel caliber as assessed on catheterization angiography and cross-sectional imaging. In patients with multiple collaterals, the presence of a dominant collateral vessel and additional accessory collaterals was recorded.

#### 2.5.4. Interventions

Patients were managed according to institutional practice based on clinical status, hemodynamic findings, anatomical characteristics, and the era of presentation. Catheter-based interventions included embolization of systemic arterial collaterals, balloon angioplasty, and stent placement within the stenosed distal anomalous pulmonary vein. Balloon angioplasty and stenting of juxta-ductal coarctation of the aorta were performed when indicated.

Surgical interventions included repair of anomalous pulmonary venous drainage and correction of associated cardiac or thoracic anomalies. The timing and type of intervention were individualized and discussed in departmental multidisciplinary meetings.

These interventions primarily included embolization of aortopulmonary collateral arteries, balloon angioplasty and stent placement for venous stenosis, as well as surgical repair of anomalous pulmonary venous drainage and correction of associated cardiac or thoracic anomalies. In selected cases, arterial interventions such as balloon angioplasty and stenting for coarctation of the aorta were also performed.

#### 2.5.5. Outcome Assessment

Clinical outcome status at last follow-up was determined based on symptom status and documented survival. Patients were classified as asymptomatic or symptomatic according to clinical documentation. 

Surgical outcome was assessed using postoperative clinical findings, imaging evaluation, and the need for repeat intervention. Reintervention was defined as any repeat surgical or catheter-based procedure performed to address recurrent or progressive venous obstruction following initial treatment.

#### 2.5.6. Functional Assessment

Functional pulmonary assessment included cardiopulmonary exercise testing (CPET) and spirometry when available. Exercise capacity was classified as normal or reduced according to age- and sex-adjusted reference values. Spirometry results were categorized as restrictive, obstructive, or normal using standard criteria.

#### 2.5.7. Follow-Up

Follow-up consisted of serial clinical assessments and imaging studies performed as clinically indicated. Follow-up duration was calculated from the time of initial presentation to the most recent clinical review.

#### 2.5.8. Statistical Analysis

Statistical analysis was performed using descriptive statistical methods. Continuous variables are reported as mean (range) or median (interquartile range [IQR] and range), as appropriate. Categorical variables are presented as frequencies and percentages.

## 3. Results

### 3.1. Demographic Characteristics

A total of 104 patients were included in the study. There was a female predominance, with 63 females (60.6%) and 41 males (39.4%). The median age at first presentation was 0.41 years (IQR 0.0–16.7; range 1 day–68 years). The median age at last follow-up was 18.5 years (IQR 8.7–30.6; range 60 days–70 years), with median follow-up duration of 9.5 years (IQR 3.7–16.1; range 58 days–38.8 years).

More than half of the cohort presented during infancy: 59 patients (56.7%) were first evaluated before 1 year of age. Within this subgroup, the median age at presentation was 17 days (IQR 1.5–75 days; range 1–330 days). Among these patients, 32 (54.2%) were female and 27 (45.8%) were male. Scimitar syndrome was diagnosed antenatally in 27 patients (25.9% of the cohort).

Demographic characteristics are summarized in Table 1.

### 3.2. Symptoms at Presentation

At initial presentation, 18 patients (17.3%) were asymptomatic. Among symptomatic patients, respiratory-type manifestations predominated. The most common presenting symptoms were shortness of breath in 51 patients (49.0%), tachypnoea in 31 patients (29.8%), recurrent chest infections in 25 patients (24.0%), exercise intolerance in 23 patients (22.1%), wheeze in 22 patients (21.2%), and respiratory distress in 19 patients (18.3%).

Cardiovascular symptoms were less frequent and included tachycardia in 12 patients (11.5%) and chest pain in 8 patients (7.7%). Hemoptysis at presentation occurred in 1 patient (1.0%).

### 3.3. Clinical Status at Last Follow-Up

At last follow-up, 44 patients (42.3%) were asymptomatic. Of these, 12 patients (27.3%) had undergone both surgical and transcatheter interventions, 10 (22.7%) surgery alone, and 7 (15.9%) transcatheter intervention alone, and 15 patients (34.1%) remained asymptomatic without any intervention.

Among patients who remained symptomatic, the most common residual symptoms were tachypnoea in 26 patients (25.0%), shortness of breath in 24 (23.1%), and exercise intolerance in 23 (22.1%). Less frequent symptoms included asthma in 16 patients (15.4%), chest pain in 10 (9.6%), recurrent chest infections in 9 (8.7%), tachycardia in 5 (4.8%), respiratory distress and wheeze each in 4 patients (3.8%), and chylothorax, syncope, and haemoptysis each in 3 patients (2.9%). At last documented follow-up, 101 of 104 patients (97.1%) were alive. Survival status beyond the last recorded clinical visit could not be confirmed for all patients, particularly those referred from abroad or lost to follow-up. Due to the retrospective design, heterogeneous follow-up duration, and incomplete longitudinal data, formal survival analysis was not performed.

### 3.4. Cardiac Position and Associated Anomalies

Abnormal cardiac position was common. Dextroposition was observed in 76 patients (73.1%), while mesocardia and levocardia were present in 21 (20.2%) and 7 patients (6.7%), respectively.

Associated cardiac anomalies were frequently identified. Secundum atrial septal defect was the most common associated lesion, present in 37 patients (35.6%), followed by persistent left superior vena cava in 20 (19.2%) and patent ductus arteriosus in 18 patients (17.3%). No additional cardiac anomaly was detected in 23 patients (22.1%). Less common associated findings included patent foramen ovale in 15 patients (14.4%), ventricular septal defect in 11 patients (10.6%), aberrant right subclavian artery in 8 patients (7.7%), coronary artery anomalies in 5 patients (4.8%), aortic coarctation in 5 patients (4.8%), right aortic arch in 4 patients (3.8%), and arch hypoplasia in 4 patients (3.8%). 

### 3.5. Pulmonary Venous Drainage Pattern 

Pulmonary venous return demonstrated marked anatomical heterogeneity across the cohort. Partial anomalous pulmonary venous return of the right lung represented the predominant configuration and was observed in 87 patients (83.6 %). Total anomalous pulmonary venous return of the right lung was identified in 17 patients (16.3%). One patient (1.0%) had left-sided partial anomalous pulmonary venous drainage to the superior vena cava.

**Figure 1:**
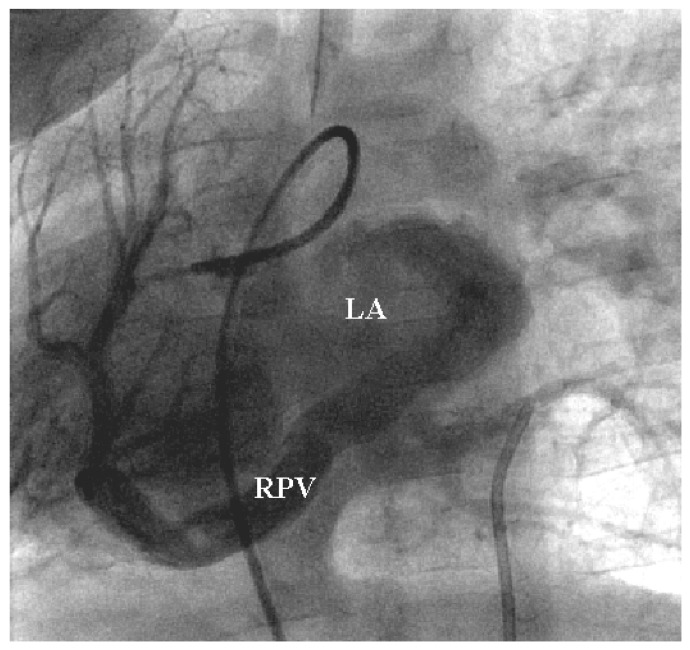
This is the laevophase of a right pulmonary arteriogram in an anterior projection showing a meandering right pulmonary vein (RPV) connecting to the morphologically left atrium (LA) in an example of a Scimitar variant.

Regarding the site of anomalous venous connection, the inferior vena cava (IVC) was the most frequent drainage location, identified in 54 patients (51.9%). In a substantial proportion of patients, the anomalous pulmonary vein connected to the IVC at or near its junction with the right atrium, a pattern observed in 31 patients (29.8%). Less common drainage sites included the superior vena cava (SVC) in 7 patients (8.7%), the right atrium in 5 patients (4.8%), the superior vena cava–right atrial junction in 3 patients (2.9%), and the azygos vein in 2 patients (1.9%). Drainage to the hepatic vein was observed in one patient (1.0%). An example of venous connection is shown in Figure 1.

### 3.6. Pulmonary and Systemic Venous Stenosis

Pulmonary and associated systemic venous stenosis (including the pulmonary veins, the scimitar vein, and the inferior vena cava [IVC]) was identified in 22 patients (21.2%) at any time during the study period. Of these, 14 patients (13.5%) had stenosis documented prior to surgical intervention, while 8 patients (7.7%) developed stenosis during follow-up after surgery.

Stenosis most frequently involved the right pulmonary veins (n = 10; 4 mild and 6 severe) and the scimitar vein (n = 8; 3 mild and 5 severe). Left pulmonary vein stenosis was present in 2 patients (1 mild and 1 severe), and IVC stenosis in 2 patients. 

**Figure 2:**
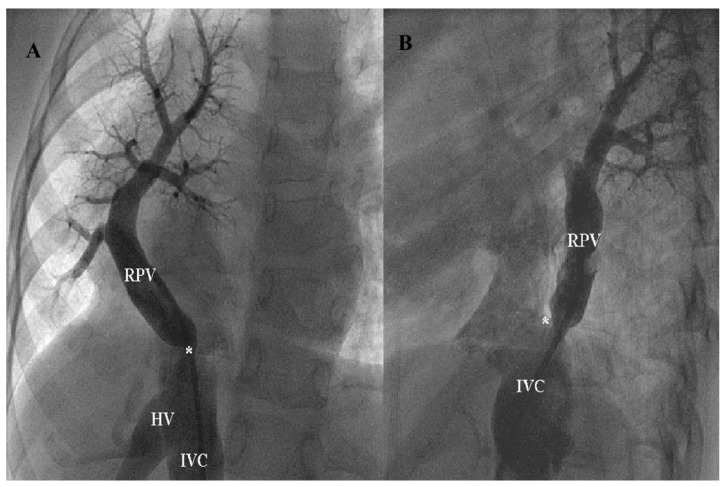
Anterior (**A**) and lateral (**B**) projections of a selective angiogram in an anomalous right pulmonary vein (RPV) that connects to the inferior vena cava (IVC) at its junction with the right atrium above the hepatic veins (HV). There is a severe stenosis (*) at its inferior part.

Postoperative stenosis involved the surgically redirected pulmonary venous pathway to the left atrium and was identified during follow-up using echocardiography, cross-sectional imaging (CMR and/or CT), and, where clinically indicated, catheter angiography. An angiographic example of severevenous stenosis is shown in Figure 2A,B.

### 3.7. Interventions and Reinterventions for Venous Stenosis

Catheter-based interventions for venous stenosis included balloon angioplasty of the scimitar vein in two patients (1.9%), balloon angioplasty of pulmonary veins in two patients (1.9%), and (IVC) stenting in one patient (1.0%). Repeat surgical intervention due to recurrent or progressive venous stenosis was required in two patients (1.9%).

### 3.8. Pulmonary Morphological Findings Pulmonary Artery Findings

**Figure 3:**
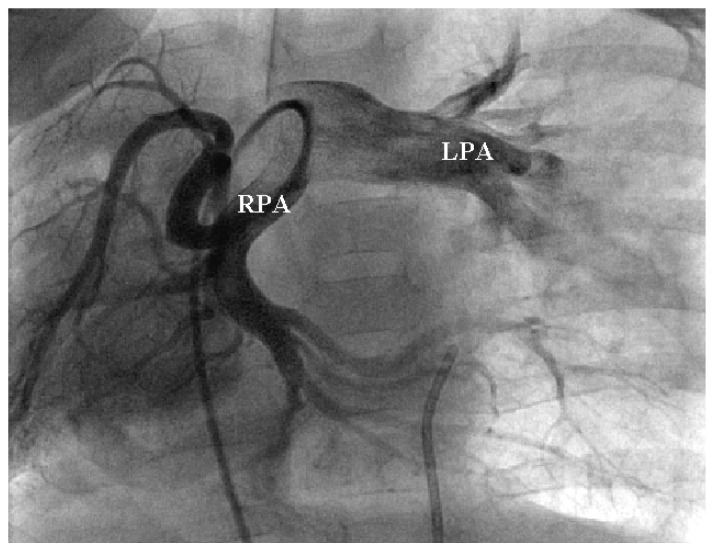
Selective right pulmonary arteriogram that clearly shows an abnormal branching pattern of the right pulmonary artery (RPA) with inferior branches crossing the midline to the left lung, suggesting a horseshoe arrangement. There is a large left main pulmonary artery (LPA).

Pulmonary artery abnormalities were frequently observed and predominantly involved the right pulmonary artery. Mild hypoplasia of the right pulmonary artery was identified in 42 patients (40.4%), while moderate-to-severe hypoplasia was observed in 33 patients (31.7%). Normal right pulmonary artery size was present in 25 patients (24.0%). Complete absence of the right pulmonary artery was rare and occurred in 4 patients (3.8%). In contrast, involvement of the left pulmonary artery was uncommon, with hypoplasia identified in only 1 patient (1.0%). A representative example of this abnormal pulmonary arterial branching pattern is demonstrated in Figure 3.

### 3.9. Pulmonary Parenchymal Findings

Pulmonary parenchymal abnormalities demonstrated considerable heterogeneity within the cohort. Mild lung hypoplasia was present in 47 patients (45.2%), affecting the right side in 37 patients (35.6%). These abnormalities were frequently associated with reduced lung volume on cross-sectional imaging (CT and/or CMR), as described in radiology reports.

Horseshoe lung was observed in 6 patients (5.8%).

### 3.10. Pulmonary and Functional Assessment 

Functional pulmonary assessment was available in a subset of patients. CPET was performed in 25 patients (24.0%); exercise capacity was normal in 15 patients (60.0%) and reduced in 10 patients (40.0%).

Spirometric evaluation was performed in 28 patients (26.9%). A restrictive ventilatory pattern predominated and was identified in 18 patients (64.3%), while obstructive and normal patterns were each observed in 5 patients (17.9%).

### 3.11. Aorto-Pulmonary Collaterals 

APCs were identified in 70 of 104 patients (67.3%), while no detectable collaterals were present in 34 patients (32.7%). Among patients with collaterals, a single dominant vessel was most common; however, multiple collaterals were present in 30 patients (28.8%). Collaterals were classified as large in 46 patients (44.2%) and small in 34 patients (32.7%), with dominant vessels typically accompanied by one or more smaller accessory collaterals.

**Figure 4:**
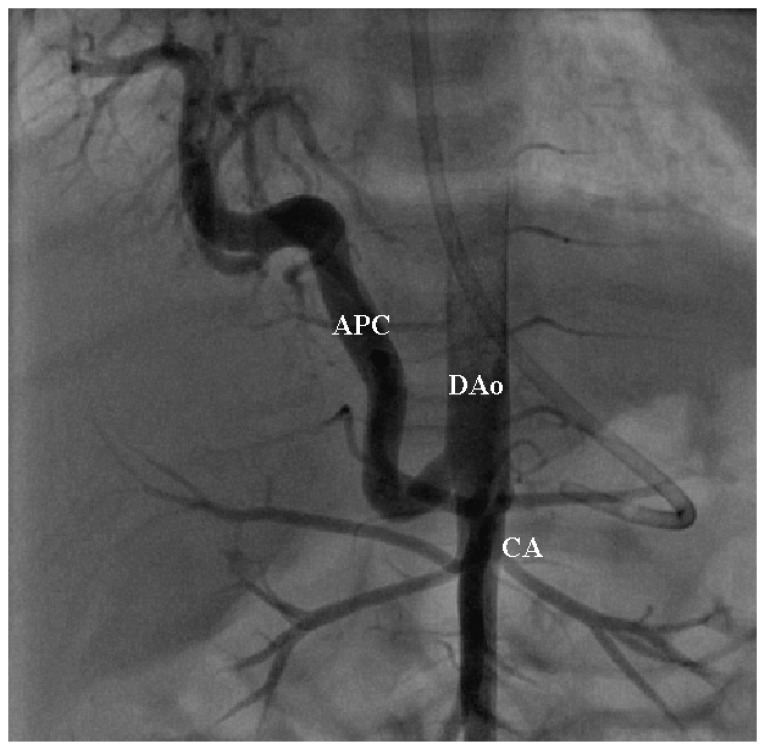
Selective descending aortogram showing a large right-sided pulmonary collateral (APC) arising from the descending aorta (DAo) above the Celiac axis (CA).

The origin of APCs was predominantly subdiaphragmatic, most commonly arising from the celiac axis in 63 patients (90%). Less frequent origins included the splenic artery in 3 patients (4.3%), the thoracic aorta in 2 patients (2.9%), and the left subclavian, right renal and hepatic arteries in 1 patient each (1.4%). A representative angiographic example of an aorto-pulmonary collateral is shown in Figure 4.

### 3.12. Surgical Management 

Surgical intervention was performed in 47 of 104 patients (45.2%) during follow-up with 12 patients (11.5%) requiring more than one surgical procedure.

Corrective surgery consisted of repairing anomalous pulmonary venous drainage, including scimitar vein repair when present, and was performed according to standard institutional practice. Among patients undergoing corrective surgery, partial anomalous pulmonary venous drainage (PAPVD) repair was performed in 27 patients (57.4%), and the Warden procedure was performed in 3 patients (6.4%). 

In addition, 27 patients (57.4%) underwent other cardiac or thoracic surgical procedures. These included atrial septal defect closure in 6 patients (22.2%), arterial duct ligation in 5 patients (4.8%), coarctation repair in 4 patients (3.8%), lobectomy in 3 patients (2.7%), ventricular septal defect closure in 3 patients (2.7%), coronary artery reimplantation in 2 patients (1.9%), diaphragmatic hernia repair in 2 patients (1.9%), and mitral valve repair in 2 patients (1.9%). Among coronary abnormalities, one patient had a fistulous communication between the left circumflex artery and the right upper pulmonary artery. Three patients demonstrated an anomalous origin of the circumflex artery from the pulmonary artery. Two of these patients underwent surgical reimplantation of the circumflex artery at the ages of 3 months and 10 months, respectively. In addition, one patient was found to have a long myocardial bridge involving the mid-left anterior descending (LAD) artery. The remaining three patients with coronary artery anomalies did not undergo any surgical or transcatheter intervention and were managed conservatively with clinical follow-up.

### 3.13. Cardiac Catheterization

Seventy-eight out of 104 patients (75.0%) underwent cardiac catheterization. Of these, 48 patients (46.2%) underwent a single catheterization, while 30 patients (28.8%) required multiple catheterization procedures. 

Cardiac catheterization before the age of 1 year was performed in 46 patients (44.2%), and interventional catheter-based procedures before the age of 1 year were required in 34 patients (32.7%).

A total of 122 catheterization procedures were performed during follow-up, of which 63 (51.6%) were interventional procedures, and 59 (48.4%) were diagnostic-only procedures.

As multiple interventional procedures could be performed in the same patient, the figures below represent patient-level counts and do not sum to the total number of interventional procedures. The most frequently performed interventional procedure was coil occlusion of systemic arterial collaterals, performed in 46 patients (44.2%). Other interventional procedures included Amplatzer Vascular Plug (AVP) device closure of major aortopulmonary collateral arteries embolization in 6 patients (5.8%), transcatheter patent ductus arteriosus (PDA) closure in 2 patients (1.9%), transcatheter atrial septal defect (ASD) closure in 2 patients (1.9%), transcatheter occlusion of pulmonary venous connection in 1 patient (1%), balloon dilatation of the pulmonary vein in 2 patients (1.9%), balloon dilatation of the scimitar vein in 2 patients (1.0%), IVC stenting in 1 patient and balloon angioplasty for coarctation in 1 patient (1.9%). Transcatheter occlusion of the anomalous pulmonary venous connection was performed in the presence of dual pulmonary venous drainage, after test occlusion demonstrated preserved pulmonary venous flow to the left atrium. A comprehensive overview of interventions, imaging findings, and venous complications is provided in Table 2.

### 3.14. Imaging Findings

#### 3.14.1. Cardiovascular Magnetic Resonance (CMR)

CMR was performed in 64 patients (61.5%), comprising a total of 95 CMR examinations; 22 patients (21.1%) underwent two or more CMR examinations. Of these examinations, 76 were performed for follow-up purposes and 19 for diagnostic assessment.

Among 44 surgically treated patients, 27 patients (61.3%) underwent CMR. Importantly, CMR was performed preoperatively in 7 surgical patients (15.9%) to assess hemodynamic significance and ventricular volumes prior to intervention.

**Figure 5:**
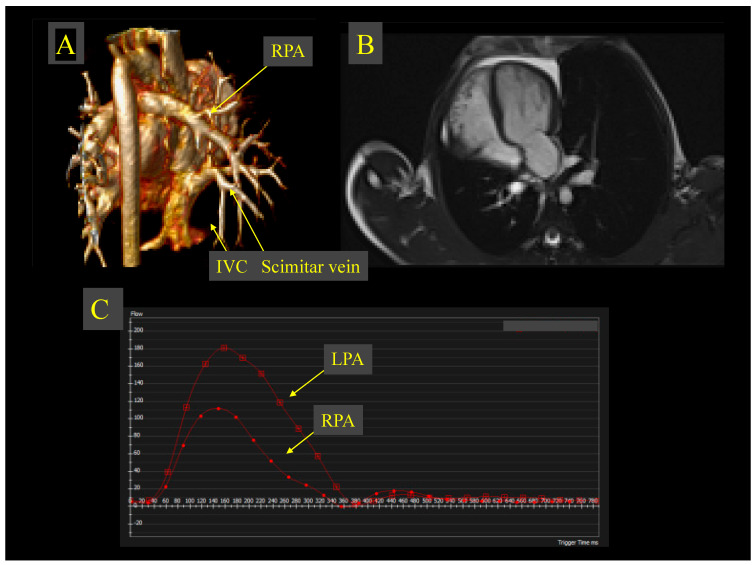
Images from a 10-year-old boy with unoperated scimitar syndrome. (**A**). 3D volume rendered CMR image reveals all right pulmonary venous drainage to the IVC via a large scimitar vein near the inferior cavo-atrial junction. (**B**). bSSFP 4-chamber view cine shows dextroposition of the heart due to hypoplastic right lung. (**C**). Flow figure from 2D phase-contrast sequences through the RPA and LPA shows significant pulmonary flow redistribution. The minority of pulmonary blood flow goes through RPA. The RPA:LPA split net flow ratio is 35:65%. The Qp:Qs ratio is 1.4:1. Indexed end-diastolic and end-systolic volumes of both ventricles were within normal range, however the ratio between right and left end-diastolic volume was increased at 1.5:1. IVC: Inferior vena cava, CMR: Cardiac magnetic resonance, bSSFP: balanced steady-state free precession, RPA: Right pulmonary artery, LPA: Left pulmonary artery, and Qp:Qs: Pulmonary-to-systemic blood flow ratio.

In patients who underwent preoperative CMR, the mean Qp/Qs ratio was 1.9 (range 1.1–2.9). The mean right-ventricular end-diastolic volume index was 107.7 mL/m^2^ (range 79–141 mL/m^2^). A representative example of multimodality CMR assessment is shown in Figure 5.

#### 3.14.2. Computed Tomography (CT)

**Figure 6:**
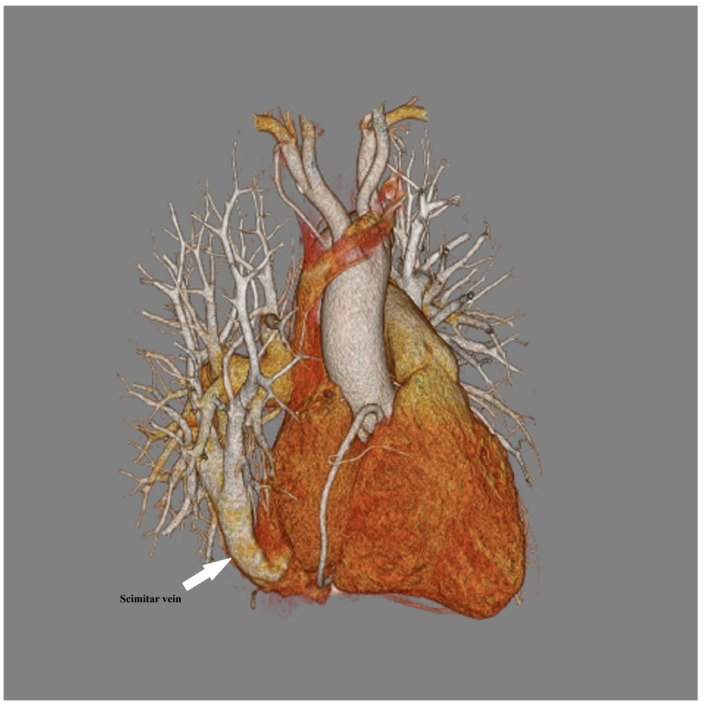
Three-dimensional computed tomography (CT) reconstruction demonstrating anomalous right pulmonary venous drainage in a patient with Scimitar syndrome. The scimitar vein (arrow) is visualized coursing inferiorly along the right cardiac border.

CT was performed in 62 of 104 patients (59.6%), comprising a total of 77 CT examinations. CT was used for diagnostic purposes in 43 patients (41.3%) and for follow-up imaging in 32 patients (30.8%). Most patients underwent a single CT examination (52 patients, 83.9%), while 10 patients (16.1%) underwent more than one CT examination; of these, 10 patients underwent at least 2 CT examinations, including 2 patients who underwent 3 CT examinations and 1 patient who underwent 4. Representative three-dimensional CT reconstructions demonstrating the characteristic scimitar vein are shown in Figure 6.

## 4. Discussion

This single-center cohort study suggests that overall survival in patients with scimitar syndrome is favorable over a median follow-up of 9.5 years. A substantial proportion of patients (44 of 104; 42.3%) were asymptomatic at last follow-up, indicating that long-term clinical stability can be achieved in many cases. However, most patients required surgical or catheter-based intervention during follow-up, reflecting the anatomical and hemodynamic complexity of the condition. Pulmonary venous and scimitar vein stenosis, including postoperative obstruction of the rerouted scimitar vein, represented an important source of morbidity and reintervention, occurring both before and after surgical repair. These findings emphasize that, despite good survival, scimitar syndrome frequently requires ongoing surveillance and individualized management. This large single-center cohort illustrates the wide anatomical, hemodynamic, and functional spectrum of scimitar syndrome and underscores the importance of an integrated, multimodality approach to diagnosis, management, and follow-up. 

Multimodality imaging plays a central role in the diagnosis and management of scimitar syndrome. It enables detailed anatomical and haemodynamic characterization, facilitates accurate identification of associated anomalies, and supports clinical decision-making regarding the need for intervention. In addition, imaging findings contribute to risk stratification and long-term follow-up, thereby influencing prognosis.

Rather than representing a uniform entity, scimitar syndrome encompasses a continuum of pulmonary venous, arterial, and parenchymal abnormalities, with clinical outcomes determined by the combined burden of these features rather than by any single anatomical characteristic. This heterogeneity is reflected in the early age at presentation observed in our cohort, in which 56.7% of patients were diagnosed within the first year of life, consistent with previous reports describing frequent early infant presentation [13]. The rate of antenatal diagnosis in our cohort was relatively modest compared with contemporary series, likely reflecting the long study period beginning in the early 1990s, preceding widespread implementation of routine fetal cardiac screening and advanced prenatal imaging.

Abnormal cardiac position was a frequent finding, reflecting the close relationship between right lung hypoplasia, reduced right thoracic volume, and cardiac displacement, as previously described within the spectrum of pulmonary venolobar anomalies [1,2]. Associated cardiac anomalies, particularly ASD, persistent left SVC, and PDA, were commonly observed and are well-recognized contributors to right-sided volume overload in this population.

Although rare, coronary artery anomalies were identified in our cohort. Anomalous origin of the circumflex coronary artery from the pulmonary artery has been reported previously in isolated cases of scimitar syndrome [13,14,15], and our findings further support the need for careful coronary assessment, as recognition of such anomalies may significantly influence surgical planning and perioperative risk.

Clinical presentation was dominated by respiratory symptoms, with cardiovascular manifestations being comparatively less frequent, consistent with previous reports describing scimitar syndrome as primarily a cardiopulmonary disorder in which respiratory morbidity often precedes overt cardiac symptoms [12,16]. Some patients were asymptomatic at presentation, highlighting the variable clinical expression of the disease and the potential for incidental diagnosis, particularly in older children and adults. During follow-up, the presence of asymptomatic patients likely reflects the combined effects of interventional, surgical, and conservative management strategies rather than the absence of underlying anatomical abnormalities. Nevertheless, a subset of patients continued to experience residual respiratory symptoms, most commonly tachypnoea, dyspnoea, and exercise intolerance, suggesting that persistent limitation may relate to pulmonary hypoplasia, abnormal pulmonary vascular development, or irreversible parenchymal changes rather than ongoing shunt physiology alone.

Pulmonary venous drainage patterns were highly heterogeneous, with partial anomalous drainage of the right lung representing the most frequent configuration. This distribution is consistent with previous series reporting partial right-sided PAPVD as the predominant form of scimitar syndrome, whereas total unilateral and left-sided variants remain relatively uncommon [1,17]. Importantly, pulmonary venous anatomy alone did not reliably predict symptom burden or the need for intervention. Instead, disease severity was more closely associated with accompanying hemodynamic factors, particularly the presence of significant aortopulmonary collaterals and pulmonary venous or scimitar vein obstruction, which have repeatedly been identified as key determinants of outcome [13,18].

Systemic aortopulmonary collaterals constitute a defining anatomical feature of scimitar syndrome and a major determinant of hemodynamic burden and clinical presentation. Previous studies have demonstrated that large or dominant collaterals may generate a substantial left-to-right shunt, occasionally reflected angiographically by a steal phenomenon from the descending aorta distal to the collateral origin, thereby contributing to pulmonary overcirculation, pulmonary hypertension, and respiratory symptoms [19,20]. In our cohort, the high prevalence of collaterals and the frequent presence of a dominant vessel, most commonly arising from the celiac axis, support the central role of collateral flow in shaping clinical severity.

Consistent with earlier series, targeted transcatheter embolization of aortopulmonary collaterals in selected patients was associated with a reduction in shunt burden and clinical improvement, without evidence of pulmonary infarction. As previously described, this is attributed to preservation of pulmonary perfusion through native pulmonary arterial branches and the bronchial circulation rather than complete dependence on collateral flow [19,21,22]. These observations reinforce that management decisions should be guided by an integrated assessment of collateral anatomy, pulmonary vascular development, and venous drainage patterns rather than by any single anatomical feature.

Pulmonary arterial abnormalities were a prominent feature in our cohort, predominantly involving the right pulmonary artery. Similar to previous reports, the spectrum ranged from mild to severe hypoplasia and, in rare cases, complete absence of the right pulmonary artery [13,21,22]. This asymmetrical pulmonary arterial development reflects impaired vascular growth of the affected lung and contributes to a reduction in the functional pulmonary vascular bed, influencing clinical presentation and long-term functional capacity, particularly when combined with systemic collaterals or pulmonary venous obstruction [13,18,21].

Pulmonary parenchymal abnormalities were also common and heterogeneous. Lung hypoplasia, most frequently affecting the right lung, was prevalent and typically associated with reduced lung volume on cross-sectional imaging. Horseshoe lung was identified in a subset of patients, consistent with previous descriptions linking this anomaly to scimitar syndrome and abnormal pulmonary vascular development and associated airway abnormalities [23,24]. These findings further support the concept that scimitar syndrome represents a spectrum of combined pulmonary vascular and lung developmental abnormalities rather than an isolated disorder of pulmonary venous drainage [23,24,25].

Pulmonary venous and scimitar vein stenosis represented one of the most clinically relevant findings in our cohort, being observed both before and after surgical intervention and remaining a major cause of reintervention. Preoperative venous stenosis was identified in a substantial proportion of patients, while newly developed or recurrent obstruction was also observed during follow-up, consistent with previous series [21,22,26]. Although overall outcomes were favorable, postoperative venous stenosis highlights that surgical correction of anomalous pulmonary venous drainage does not fully eliminate the risk of progressive or recurrent obstruction. Variability in postoperative outcomes has been reported with different surgical techniques, including baffle repair and reimplantation, and appears to be influenced by patient age, anatomical complexity, and timing of surgery [24,27]. In our cohort, the number of patients undergoing Warden-type repair was limited, precluding meaningful technique-specific comparisons.

The need for a catheter or repeat surgical interventions underscores the dynamic nature of venous pathology in scimitar syndrome. Persistent or recurrent stenosis is likely influenced not only by technical aspects of repair but also by underlying pulmonary venous hypoplasia, abnormal pulmonary vascular development, and altered flow dynamics, emphasizing the importance of careful long-term surveillance with multimodality imaging irrespective of the initial treatment strategy [26,28].

Cardiac catheterization played a central role in longitudinal management, serving both diagnostic and therapeutic purposes across a wide age range. The frequent need for invasive assessment and repeated procedures reflects the complex and evolving hemodynamic substrate of scimitar syndrome, particularly in patients presenting early in life, consistent with previous reports [18,26,29]. Catheter-based intervention, most commonly the coil embolization of systemic collaterals, was frequently required during infancy, underscoring the role of early transcatheter management in reducing hemodynamic burden and facilitating staged treatment strategies [18,28].

CMR emerged as a key complementary imaging modality for functional assessment and longitudinal follow-up. Although not routinely performed at initial presentation, its use during follow-up reflects its value in the non-invasive surveillance of ventricular volumes, shunt magnitude, anatomy, evolving hemodynamic changes and residual postoperative findings. In a limited subset of surgically treated patients, preoperative CMR contributed to the assessment of surgical indication and timing, consistent with prior literature [29]. CT provided high-resolution anatomical delineation of pulmonary venous connections, systemic arterial collaterals, and lung morphology, and was particularly valuable for defining features of direct surgical relevance and associated anomalies [30,31].

Despite the high prevalence of structural pulmonary and vascular abnormalities, functional assessment revealed preserved exercise capacity in a substantial proportion of patients undergoing testing. CPET demonstrated that many patients maintained normal functional performance, while spirometry most commonly showed a restrictive ventilatory pattern when abnormal. These findings are consistent with previous reports indicating that lung hypoplasia and reduced pulmonary volume, rather than primary airway disease, are the predominant determinants of functional limitation in scimitar syndrome, particularly in childhood [32].

Overall, our findings reinforce that scimitar syndrome should be approached as a heterogeneous spectrum of cardiopulmonary malformations. Multimodality imaging is essential for accurate diagnosis, risk stratification, timing of intervention, and long-term follow-up. Particular attention should be directed toward pulmonary venous pathways and systemic arterial collaterals, as these features exert the greatest influence on clinical course and the need for reintervention. Long-term surveillance remains crucial, even in asymptomatic patients, given the potential for late-onset venous obstruction and evolving functional impairment.

Each imaging modality has specific advantages and limitations. Echocardiography is widely available and useful for initial assessment, CT provides detailed anatomical delineation, and CMR allows comprehensive functional evaluation without ionizing radiation. However, each modality has inherent limitations, and a multimodality approach is often required for accurate diagnosis and optimal management [8,33]. Each modality provides complementary information, and no single technique is sufficient for complete evaluation.

This study is limited by its retrospective design and the long study period, which may introduce heterogeneity in clinical management and follow-up. In addition, measurements were not obtained at standardized time points, reflecting real-world clinical practice. Despite these limitations, this study represents one of the largest single-center cohorts with long-term follow-up in patients with scimitar syndrome.

## Figures and Tables

**Table 1:** Clinical Presentation and Anatomy.

Category	Finding	n (%)
Demographics	Female sex	63 (60.6%)
	Presentation < 1 year	59 (56.7%)
Cardiac Position	Dextroposition	76 (73.1%)
Associated CHD	ASD	37 (35.6%)
	Persistent left SVC	20 (19.2%)
	PDA	18 (17.3%)
	Any coronary anomaly	5 (4.8%)
	No associated CHD	23 (22.1%)
Venous Drainage	Partial anomalous pulmonary venous drainage (PAPVD)	87 (83.7%)
	Total right lung drainage	17 (16.3%)
	Drainage site: IVC ± RA junction	85 (81.7%)
Lung & PA Anatomy	Right PA hypoplasia (any)	74 (71.1%)
	Lung hypoplasia (any)	84 (80.8%)
	Horseshoe lung	6 (5.8%)
Symptoms	Symptomatic at first review	86 (82.7%)
	Asymptomatic at last follow-up	44 (42.3%)
Functional Tests	Normal CPET	15/25 (60.0%)
	Restrictive spirometry	18/28 (64.3%)

Abbreviations: ASD = atrial septal defect; CHD = congenital heart disease; CPET = cardiopulmonary exercise testing; IVC = inferior vena cava; PA = pulmonary artery; PDA = patent ductus arteriosus; RA = right atrium; and SVC = superior vena cava.

**Table 2:** Interventions, Imaging Findings, and Venous Complications.

Domain	Key Finding	n (%)
Aorto-pulmonary collaterals	Present	70 (67.3)
	Multiple collaterals	30 (28.8)
Cardiac catheterization	Any catheterization	78 (75.0)
	Total procedures	122
	Interventional procedures	63 (51.6)
Surgery	Any surgical intervention	47 (45.2)
Venous stenosis	Any venous stenosis	22 (21.2)
	Pulmonary/scimitar vein stenosis	20 (19.2)
	IVC stenosis	2 (1.9)
	Preoperative stenosis	14 (13.5)
	Postoperative stenosis	8 (7.6)
Imaging	Any CMR	64 (61.5)
	Any CT	62 (59.6)

Abbreviations: CMR = cardiovascular magnetic resonance; CT = computed tomography; nd IVC = inferior vena cava. Total number of catheter-based procedures performed; percentages are not provided because multiple procedures were performed in some patients.

## Data Availability

The datasets generated and/or analyzed during the current study are available from the corresponding author on reasonable request and are not publicly available due to patient confidentiality.

## Abbreviations

APC	Aorto-pulmonary collateral
ASD	Atrial septal defect
CMR	Cardiovascular magnetic resonance
CPET	Cardiopulmonary exercise testing
CT	Computed tomography
IVC	Inferior vena cava
LAD	Left anterior descending coronary artery
PAPVD	Partial anomalous pulmonary venous drainage
PDA	Patent ductus arteriosus
Qp/Qs	Pulmonary-to-systemic flow ratio
SVC	Superior vena cava
